# Galectins and Ovarian Cancer

**DOI:** 10.3390/cancers12061421

**Published:** 2020-05-31

**Authors:** Chisa Shimada, Rui Xu, Linah Al-Alem, Marina Stasenko, David R. Spriggs, Bo R. Rueda

**Affiliations:** 1 Department of Obstetrics and Gynecology, Vincent Center for Reproductive Biology, Massachusetts General Hospital, Boston, MA 02114, USA; cshimada@mgh.harvard.edu (C.S.); rxu9@mgh.harvard.edu (R.X.); lal-alem@mgh.harvard.edu (L.A.-A.); dspriggs@mgh.harvard.edu (D.R.S.); 2 Obstetrics, Gynecology, and Reproductive Biology, Harvard Medical School, Boston, MA 02115, USA; 3 Gynecology Service, Memorial Sloan Kettering Cancer Center, 1275 York Ave, New York City, NY 10065, USA; stasenkm@mskcc.org; 4 Department of Hematology/Medical Oncology, Massachusetts General Hospital, Boston, MA 02114, USA; 5 Division of Gynecologic Oncology, Department of Obstetrics and Gynecology, Massachusetts General Hospital, Boston, MA 02114, USA

**Keywords:** ovarian cancer, galectins, invasion, metastasis, chemoresistance, immune suppression

## Abstract

Ovarian cancer is known for its aggressive pathological features, including the capacity to undergo epithelial to mesenchymal transition, promoting angiogenesis, metastatic potential, chemoresistance, inhibiting apoptosis, immunosuppression and promoting stem-like features. Galectins, a family of glycan-binding proteins defined by a conserved carbohydrate recognition domain, can modulate many of these processes, enabling them to contribute to the pathology of ovarian cancer. Our goal herein was to review specific galectin members identified in the context of ovarian cancer, with emphasis on their association with clinical and pathological features, implied functions, diagnostic or prognostic potential and strategies being developed to disrupt their negative actions.

## 1. Introduction

**Figure 1:**
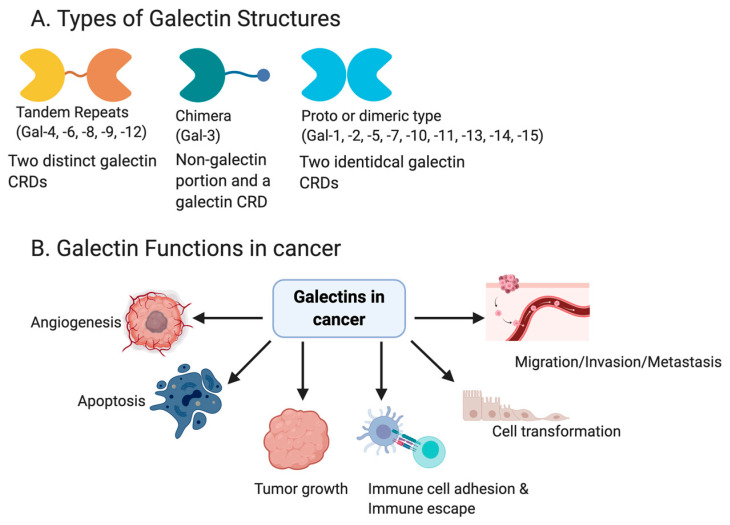
A schematic showing the different galectin structures and members of each (**A**). Galectins have been shown to play a role in altering many functions in cancer, including angiogenesis, apoptosis, tumor growth, immune escape, immune cell adhesion, cell transformation and metastasis/invasion (**B**).

Recognizing the importance of the biological information dictated by cell surface carbohydrates, investigators found it necessary to isolate and characterize the glycoconjugates responsible for complex cell-surface glycan interactions. Galectins are a family of endogenous lectins defined as small, soluble β-galactoside binding proteins [1], which were first discovered in 1975 in the electric eel [2,3]. As of today, there are 16 galectin family members in mammals, which are numbered based on their order of discovery. However, some galectins identified in mammals were not found in humans. These include Galectin-5 (Gal-5), which has only been identified in rats, Galectin-6 (Gal-6) in mouse, Galectin-11 (Gal-11), and Galectin-15 (Gal-15) in sheep and goat [4,5]. The galectin family members are classified into three subfamilies, based on the structure and number of carbohydrate recognition domains (CRD) that they possess (Figure 1A). The CRD is a stretch of ~130 conserved amino acids [6]. The largest subfamily are homodimers, which include Gal-1,-2,-5,-7,-10,-11,-13, -14,-15, and contain a single CRD. The second subfamily are galectins with tandem-repeats, which include Gal-4,-6,-8,-9,-12. These galectins, in contrast to the homodimeric subfamily, contain double and non-identical CRD in a single polypeptide chain. Lastly, one of the galectins, Gal-3, is under the chimera subfamily. Galectin-3 contains one CRD connected to a non-lectin domain, capable of forming a multimeric structure [7].

### 1.1. General Role of Galectins

Galectins are commonly synthesized on free polyribosomes and primarily present in the cytoplasm, but are often shuttled between the cytoplasm and nucleus. Galectins are also expressed on the cell surface and in the extracellular matrix. Intracellularly, galectins are involved in the regulation of apoptosis and proliferation, as well as mediate pre-mRNA splicing [8]. The presence of galectins within and on the extracellular matrix of the cell allows them to mediate cell-to-cell and cell-to-matrix interactions, which depend on the glycosylation patterns of the cells. Those glycosylation patterns, in turn, reflect the underlying nutritional state of the microenvironment and availability of carbohydrate precursors. Although this review will focus primarily on the role of Gal-1, Gal-3, and Gal-7 in ovarian cancer, a brief description of some of the nonmalignant functions of these and other galectins is included below.

### 1.2. Galectin Mediated Immune Functions

Galectins are expressed on cells of the innate immunity (dendritic cell, macrophages, mast cells, natural killer cells, gamma/delta T cells, and B-1 cells). They are also expressed on members of the adaptive immunity (activated B and T cells) [9,10]. Their ability to regulate the innate and adaptive immune system has been extensively described in the literature [11,12,13]. Galectins appear to have a key role in the cell-cell interactions in inflammation. As an example, Gal-1 can act as a potential immunosuppressive agent, leading to a restoration of immune cell homeostasis in settings of autoimmunity and inflammation [14,15,16].

Additionally, Toscano et al. reported that recombinant Gal-1 suppressed retinal inflammatory disease by promoting T_reg_ cell-mediated anti-inflammatory response [17]. Other examples include the role of Gal-3 in IL-2-dependent T cell growth [18] and Gal-9 activating inflammatory cytokine gene expression by interacting with NF-IL6 in THP-1 cells [19]. In an animal model of arthritis, *Lgals3*^-/-^ mice were found to develop less joint inflammation, less bone erosion, and lower amounts of IL-17 producing cells in the spleen, compared to their wild type counterparts [20].

### 1.3. Galectins and Cancer

Galectins are distributed in a cell-specific manner and are often differentially expressed in tumor cells relative to the normal cells [21]. The aberrant regulation of galectins have been implicated in several cancer types, including head and neck [22], gastric [23,24], colorectal [25,26], bladder [27], melanoma [28,29], and gynecological cancers [30,31,32]. The function of galectins (Figure 1B) differs depending on the type of cancer, as well as whether the specific galectins are intercellular or extracellular. Functions such as apoptosis have been linked to Gal-1 [33], Gal-3 [34,35], Gal-7 [36,37] and Gal-9 [38]. However, galectins have had opposing functions described in different cancers. For example, Gal-1 induced apoptosis in prostate [33] and colon cancer [39], but had anti-apoptotic effects in cervical [40] and lung cancers [41].

Similarly, the differential regulation of apoptosis was seen with Gal-3 [35,42,43,44] and Gal-7 [36,45]. Because galectins are found in the extracellular space, the cellular surface, the cytoplasm, and even in the nucleus, the location of Galectin proteins drives aspects of biological function. For instance, Gal-3 induced tumor growth, angiogenesis, and reduced apoptosis when present in the cytoplasm, and had anti-tumor effects when it is nuclear [46].

### 1.4. Ovarian Cancer and the Implication of Galectins in Its Pathology

Ovarian cancer is the most lethal of the gynecologic cancers in the United States [47]. Its lethality is due in a large part to its aggressive features and inability to be diagnosed at an early stage. Hence, the bulk of patients present, at a late stage, with a highly metastatic, invasive disease. Ovarian cancer is highly heterogeneous, with adenocarcinoma making up the bulk of the malignancies [48,49]. The most common histophenotype is high-grade serous ovarian cancer with the endometrioid, clear cell and mucinous subtypes being less prevalent. The current treatment strategy includes surgical debulking followed by a regimen of a platinum and taxane based chemotherapy, or alternatively, neoadjuvant chemotherapy with interim cytoreduction followed by additional cycles of chemotherapy. Unfortunately, the greater majority of the patients will present with recurrent platinum resistant disease in 36 months [50,51,52,53,54]. Many of the aggressive features of ovarian cancer are likely attributed, at least in part, to one or more of the pro-tumor galectin family members. Herein, we provide an overview of LGALS gene expression, the levels and locality of specific galectin proteins, their regulation at the mRNA and/or protein levels, and their potential functional role as it pertains to the genesis, progression, and overall pathology in ovarian cancer.

## 2. Galectin-1 (LGALS1)

### 2.1. General Functional Roles of Galectin-1

Galectin-1 is encoded by the human LGALS1 gene, and similar to the other galectins, is characterized by its affinity for β-galactoside-containing glycans [55,56]. Galectin-1 exists in both a homodimeric and monomeric form. The different forms are associated with different functions, which have been reviewed in detail by others [57,58]. Galectin-1 has been shown to mediate cellular functions, including cell-cell interactions, cell proliferation, cell migration, adhesion, immune cell function, cell signaling, and apoptosis [57,59,60,61,62,63]. The full spectrum of intracellular and extracellular functions of Gal-1, like many other galectins, remains to be completely defined in its diverse cell type. Moreover, the recognized functions may differ during cell proliferation, differentiation, transformation to a benign hyperplastic state, or a malignant transformation. It is also possible that aberrant galectin expression, whether it be a result of a stress-related stimulus, where it is localized within the cell or on a neighboring cell, or in response to a gain or loss of function, may be a primary driver of malignant transformation or serve to promote of a more aggressive phenotype.

### 2.2. Gal-1 Expression and Localization in Ovarian Cancer

Galectin-1 levels have been assessed in multiple tumor types [22,30,63,64,65]. In general, the majority of the studies described have shown a positive correlation with Gal-1 levels and increased tumor invasiveness and/or metastasis in areas including prostate, lung, and neuroblastoma [33,41,66,67,68].

One of the first studies describing Gal-1 in ovarian cancer demonstrated an increased Gal-1 expression relative to the normal ovary [69]. The Gal-1 positive staining was reported to be heterogenous from sample to sample. Both tumor and stromal cells showed positive staining. It was noted that in those areas where there was invasive carcinoma, the stroma was more likely to be positive for Gal-1 than in the stromal cells distal from the tumor. The authors speculated that the presence of Gal-1 in the stroma was due to the release of the lectin by the neighboring tumor cells. In situ hybridization was performed to ascertain which cells expressed Gal-1 mRNA on an additional subset of tumors [69]. Albeit a small cohort, it revealed that Gal-1 mRNA expression was found predominantly in the stroma with moderate positivity in the cancer cells themselves. Interestingly, there was no correlation with Gal-1 protein levels (based on immunohistochemistry) and their corresponding clinical pathologic features [69].

Van den Brûle et al. [69] extended their findings by evaluating the levels of Gal-1 in several ovarian cancer cell lines (AZ364, SKOV3, AZ224 OVCAR-3, AZ419, and AZ382), by Western blot analysis. Of the lines assessed, Gal-1 was only detected in the AZ364, SKOV3, and AZ224 and not in the OVCAR-3, AZ419, and AZ382 cell lines. The investigators utilized flow cytometry to assess the cell membrane levels of Gal-1, in an attempt to distinguish between intra and extracellular levels. Of those that they could assess, only the AZ364, SK-OV-3, and AZ224 cell lines had evidence of Gal-1 on the surface of the cell membrane, although it lacks a transmembrane domain and is likely bound to the glycosylation structures of cell surface proteins. Given that these investigators postulated that the Gal-1 was secreted, they also assessed the conditioned medium from their lines shown to be positive for Gal-1, suggesting that other, nonclassical mechanisms are responsible for its extracellular location. Thus, despite concentrating the media, they were unable to show evidence of Gal-1 by Western blotting techniques. This finding suggests that free Gal-1 was not passively or actively being released into the medium under the culture conditions described. Whether this would change under other conditions remains to be determined. Using the fibroblast line, 84BR, which expresses Gal-1, the authors investigated whether the secretion of Gal-1 could be modulated by conditioned medium from 6 ovarian cancer cell lines. Conditioned media from four of the six lines cultured with 84BR resulted in an increase in secreted Gal-1 [69].

### 2.3. Gal-1 Function in Ovarian Cancer

To determine whether Gal-1 influenced cell proliferation, they utilized bromodeoxyuridine (BrdU) incorporation as a readout. Recombinant Gal-1 was added to the cultures at increasing concentrations. No increase in cell proliferation was observed at the lower concentrations, and at the highest concentrations, there was a decrease in cell number in all the lines tested. It was suggested that maybe physiologically elevated Gal-1 in the stroma might serve as a defense against the invading tumor, although this explanation was not well justified. Finally, they demonstrated that the coculture with recombinant Gal-1 could enhance cancer cell binding to either fibronectin or laminin in three of the six lines, which matches the idea that Gal-1 can contribute to the invasive or migratory properties of a cell.

In a second study by Kim et al. [70], the investigators assessed a small cohort of primary tumor samples from patients diagnosed with ovarian cancer by immunohistochemistry. Of the samples evaluated, the greater majority were of the serous subtype, with only a few endometrioid and mucinous subtypes. Although Gal-1 positive staining was present in the peritumoral stroma of all tumor samples, there was no evidence of Gal-1 in the normal ovarian tissues evaluated. Of the tumor samples assessed, they were divided into two groups; a low to moderate and high expression group. After determining the clinical correlates, it was determined that the high expressors were associated with advanced-stage disease, a serous histology, and the patients had evidence of a greater residual tumor volume after initial debulking surgery. Those tumors that had lower levels of Gal-1 in the peritumoral stroma were more sensitive to fist line taxane-carboplatin treatment, whereas those that had higher levels in the peritumoral stroma were more likely to be resistant and had a poor PFS (22.5 vs. 48 months) [70]. These findings were later corroborated by Zhang and colleagues [71] who used quantitative RT-PCR, Western blot, and immunohistochemistry analyses on fresh frozen or paraffin-embedded samples to demonstrate that high Gal-1 levels were positively correlated with advanced stage and poor prognosis. When broken down into stages, the investigators found that normal ovarian tissue had little to no Gal-1 mRNA or protein. The levels of Gal-1 mRNA and protein were greater in stage III-IV than in stage I-II samples.

To assess the tumor samples via immunohistochemistry, the tumor sections were stained, scored, and divided up into low and high expressors. Based on this differentiation, they determined that the high expressors had a shorter PFS time than the low expression group.

In the Kim et al. study, the investigators assessed the functional contributions of Gal-1 in ovarian cancer, utilizing the HeyA8, A2780-CP20, and SKOV3ip1 cell lines. Galectin-1 levels were shown to be most prominent in the HeyA8 and the SKOV3ip1 lines, as evidenced by Western blot analysis. Given that Gal-1 was present in the peritumoral region of the primary tumors, human endometrial fibroblast line T HESCs were also evaluated for Gal-1 presence. Western blot analysis revealed that these cells were also positive for Gal-1. The HeyA8 and SKOV3ip1 cells were subsequently transfected with a Gal-1 siRNA. After determining that the siRNA knockdown of Gal-1 was successful, 3-(4,5-Dimethylthiazol-2-yl)-2,5-diphenyltetrazolium bromide (MTT) based assays were performed with the Gal-1 siRNA and Gal-1 control cells. The reduction in Gal-1 was concurrent with the decrease in metabolic activity, which was interpreted to be a decrease in cell proliferation. The A2780-CP20 line, which was initially determined to have a low level of Gal-1, was transfected with a recombinant Gal-1 protein, resulting in an increase in cell proliferation. The HeyA8 and SKOV3ip1 Gal-1 siRNA and the A2780-CP20 recombinant cells and their respective controls were then tested for their differential invasive properties in a Matrigel transwell system. The Gal-1 siRNA cells had a reduced invasion compared to their control. In contrast, the Gal-1 overexpressing cells displayed an increased rate of invasion relative to its control.

In response to previous findings by others, suggesting that cancer cell-associated fibroblasts promoted the invasive and migratory properties of malignant cells [72], Kim et al. assessed Gal-1 levels in the conditioned media of the T-HESCs. The levels found in the conditioned media of the siRNA cells were less than their respective controls. Although not reported, it would have been interesting to assess the effect of the Gal-1 knockdown and overexpression T-HESCs when co-cultured with the HeyA8, SKOV3ip1, and A2780-CP20 cell lines. To begin to evaluate Gal-1’s role in platinum resistance, a siRNA strategy was used to reduced Gal-1 levels in A2780CP cells. The reduced Gal-1 levels corresponded with an increase in cells undergoing apoptosis. In contrast, forced expression of Gal-1 in Hey cells made them more resistant to cisplatin.

**Figure 2:**
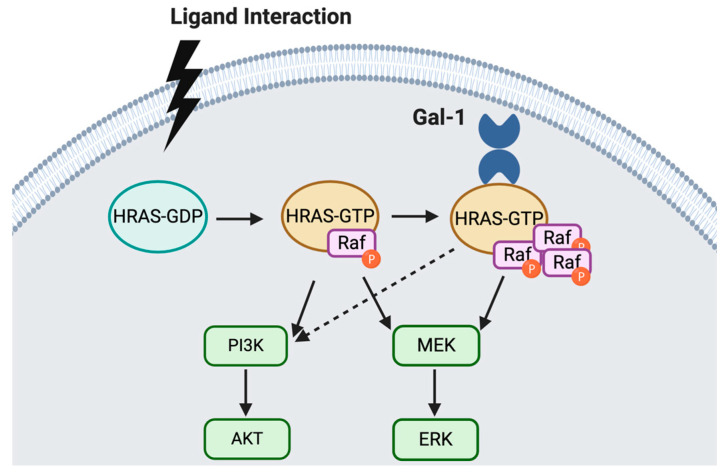
Oncogenic H-Ras plays a major role in tumor transformation via two major pathways, PI3K/AKT and MEK/ERK [73,74]. H-Ras recruits intracellular Gal-1 from the cytosol. This interaction enhances H-Ras-mediated cell transformation. Since Gal-1 has no effect on the membrane localization of inactive H-Ras, Ras activation, via GTP binding is needed for the H-Ras/Gal-1 interaction. Gal-1 is then able to enhance H-Ras-GTP, leading to an increase in Raf-1 recruitment, which culminates in a sustained activation of the MEK-ERK pathway and enhanced cell transformation [63].

Similar functional studies were performed by Zhang et al., whereby they also utilized siRNA techniques to assess the impact of reduced Gal-1 on cell proliferation, migration, and invasion in vitro. Of interest, they evaluated the levels of Gal-1 in SKOV3, CAOV3, SKOV3ip1, Hey, and A2780cp cell lines. In contrast to the study by Kim et al., the A2780cp Gal-1 levels were similar to SKOV3, CAOV3, and SKOV3ip1, and the Gal-1 levels in the Hey cells were low. Nevertheless, they showed that the siRNA induced reduction of Gal-1 in SKOV3ip1 cells corresponded with reduced cell proliferation and invasion. Unlike the transient transfection of recombinant Gal-1 in the Kim study, Zhang and colleagues used a lentiviral-based strategy to overexpress Gal-1 in the Hey cell line. The increase in Gal-1 was concurrent with increased cell proliferation rate, as determined by CCK-8 assay. Using the transwell invasion/migration assay, they further demonstrated that the rise in Gal-1 expression increased Hey cell proliferation, migration, and invasion, relative to their corresponding controls. Given the previously described role that Gal-1 had in facilitating membrane-associated Ras [40,41], Zhang et al. demonstrated that the downregulation of Gal-1 decreased H-Ras, p-Raf-1, and p-ERK expression. In contrast, increased Gal-1 expression corresponded with increased H-Ras, p-Raf-1, and p-ERK expression. This suggests a sustained activation of theses pathways, as depicted in Figure 2. Furthermore, using co-immunoprecipitation, they demonstrated that Gal-1 and H-Ras interacted; whether it was direct or indirect interaction, was not shown. Therefore, Gal-1 may interact with H-Ras to activate the ERK pathway and promote epithelial ovarian cancer pathology by promoting cell invasion and proliferation [71].

Galectin-1 knockdown was previously reported to sensitize lung cancer cells to platinum-based chemotherapy [41]. There have since been similar reports in in vitro and in vivo models of ovarian cancer [71,75]. Zhang and colleagues used an siRNA-based strategy to down-regulate Gal-1 levels in cisplatin-resistant ovarian cancer cells (A2780CP cells) [71]. Following treatment with cisplatin, they found that cell proliferation was inhibited, and levels of apoptosis increased in a dose-dependent manner, relative to the controls. Interestingly, the cisplatin-resistant ovarian cancer cells transfected with Gal-1 siRNA were more responsive to the negative effects of cisplatin than those transfected with the control siRNA. These findings [71], and those of others [55,69,70,76], suggest that if a targeted disruption of Gal-1 expression/function in tumor tissue or the immediate tumor microenvironment could be obtained, it may be a plausible therapeutic option for cisplatin-resistant ovarian cancer.

As discussed, previous studies of non-ovarian tumor types provided evidence to support the concept that Gal-1 promotes cancer cell invasion and/or metastasis, processes mediated, at least in part, by c-jun-NH2-terminal kinase 1 (JNK1) signaling [77,78]. Moreover, there is data that supports the idea that JNK1 signaling is associated with an epithelial-mesenchymal transition (EMT) [79,80,81]. Epithelial-mesenchymal transition is a critical process that often contributes to the invasive and metastatic potential of tumor cells [82]. Consequently, Zhu and colleagues investigated the relationship between Gal-1 and EMT in ovarian cancer. To accomplish this, they began by comparing the levels of Gal-1 and E-cadherin in a cohort of 107 samples, from patients diagnosed with ovarian cancer of varied histologies. It is important to note that this cohort had high- and low-grade serous cancer samples, as well as other low and high-grade histologies. The investigators reported that the higher levels of Gal-1 were closely associated with higher grade, more lymphatic metastases, and advanced stage. In addition, they determined that there was a negative correlation between Gal-1 and E-cadherin.

Given the limited knowledge of how Gal-1 might mediate the enhancement of EMT in ovarian cancer, Zhu et al. [56] utilized the SKOV3ip and SKOV3 cell lines that were transfected with Gal-1 siRNAs or transduced with a Gal-1 lentivirus. Similar to other studies described above [70,71,75], they assessed basal Gal-1 levels in several ovarian cancer cell lines, A2780cp, A2780, SKOV3, SKOV3ip, and Hey cells. The SKOV3ip cell line demonstrated the highest levels of Gal-1, while their SKOV3 cells displayed the lowest level of the group. Armed with this information and the knowledge that galectin mediated functions are often dictated by their cellular location (cytosolic, nuclear, etc.) the investigators took the initiative to define the location of the Gal-1 in the two cell lines. In this case, both lines demonstrated a cytosolic presence. The subsequent decrease in Gal-1 in the SKOV3ip induced by the siRNAs corresponded with the reduction in invasive and migratory abilities of these cells, in a similar way to that seen by others [69,70,71]. Similarly, the expression increased as a result of the lentiviral infection of Gal-1 enhanced invasive and migratory activity of the SKOV3 control cells. To begin to explore whether these changes were the result of intracellular or secreted Gal-1, Zhu et al. used a Gal-1 antibody to ascertain if it would block the invasive or migration. Upon seeing no change, they speculated that the effect of Gal-1 was the result of intracellular mediated action.

They further demonstrated that the downregulation of Gal-1 with a siRNA-based strategy resulted in an increase in levels of mRNA for E-cadherin and decreased levels of N cadherin, MMP7, uPA, and fibronectin snail and slug. The changes in *E-cadherin* and *N-cadherin* at the mRNA level corresponded to what was observed at the protein level. As anticipated, the opposite was found in the SKOV3 Gal-1, whereby overexpressing cells demonstrated decreased levels of mRNA encoding *E-cadherin* with an increase in mRNA for N-cadherin, MMP7, uPA and fibronectin Snail and Slug. Again, the changes in E- and N- cadherins reflected that of the changes observed at the mRNA level. Collectively, these results led the investigators to conclude that Gal-1 played a significant role in the EMT- mesenchymal epithelial transition (MET) plasticity of ovarian carcinoma cells [56].

Based on previous studies suggesting that activated MAPK JNK/p38 signaling pathway could contribute to EMT in malignant tumors [83], Zhu et al. [56] assessed whether this pathway was involved in the regulation of EMT by Gal-1 in their siRNA Gal-1 knocked down SKOV3ip cells and Gal-1 overexpressing SKOV3 cells. The Gal-1 siRNA cells demonstrated a decrease in the basal phosphorylation levels of phosphorylated JNK and p38 in the siRNA Gal-1 knocked SKOV3ip cell line. Conversely, the lentivirus transduction of Gal-1 in SKOV3ip1 cells decreased the basal phosphorylation levels of MAPK JNK/p38. These data support the concept that elevated Gal-1 levels can promote EMT in ovarian cancer cells via MAPK JNK/p38 signaling.

To determine if the activation of the MAPK JNK/p38 signaling pathway was concurrent with the regulation of Gal-1 on EMT in ovarian cancer cells, they utilized a pharmacologic approach. Specifically, they used the JNK antagonist (SB203580), the JNK/p38 antagonist (SP600125), and used a MAPK JNK/p38 agonist, anisomycin. Both the antagonists reduced N-cadherin and vimentin expression in the SKOV3-Gal-1 overexpressing cells, which corresponded with an increase in E-cadherin levels. However, the Gal-1 agonist, anisomycin, decreased E- cadherin expression and upregulated N-cadherin and vimentin expression in Gal-1 siRNA- transfected SKOV3ip cells. Treatment with anisomycin enhanced cell migration and invasion properties of the Gal-1 siRNA-transfected SKOV3ip cells. In addition, both SB203580 and SP- 600125 decreased migration and invasion properties in the SKOV3 Gal-overexpressing cells. Interestingly, these effects are evident in their respective controls. Nevertheless, their overall response to the agonists and antagonists provides evidence to support the potential importance of the MAPK JNK/p38 signaling in Gal-1 mediated EMT and metastasis in ovarian cancer.

The contribution of MAPK JNK/p38 signaling to metastasis was affirmed in an in vivo model of ovarian cancer. Using the nude mouse tumor model, Gal-1 upregulation promoted the metastasis of SKOV3 cells. Importantly, treatment with antagonists of the MAPK JNK/p38 signaling pathway reduced the metastatic potential of Gal-1, overexpressing SKOV3 cells in mice [56].

At this point, it is important to note that when comparing the various studies described above or to follow, there are contrasting data related to the basal or induced galectin levels in the multiple cell lines. Whether this is a result of different cell densities, variations in cell culture conditions, or detection methods used, is not yet known. Nevertheless, the investigators involved in the different studies have, for the most part, triaged them to high and low expressors and used them accordingly in their individual model systems. Therefore, rather than focus on the contrasting aspects, we attempted to provide a conceptual perspective.

In a study by Park et al. [76], they were investigating the differences in toll-like receptor (TLR) mediated phosphoinositol 3 kinase (PI3K) signaling activity in CAOV3 and SKOV3 ovarian cancer cell lines. The investigators had postulated that Gal-1 might be a promising candidate for downstream targeting of the TLR/PI3k mediated signaling pathway in metastatic ovarian cancer. For their purposes, the CAOV3 line was supposed to represent a line from a primary tumor, whereas the SKOV3 was representative of a metastatic lineage. Of interest herein, Gal-1 levels were found to be elevated in the SKOV3 cell line when treated with a TLR4 agonist, LPS, a TLR3 agonist (poly I:C), and a TLR2/6 agonist MALP2, relative to the vehicle control. No effect was observed in the CAOV3 cell line. The TLR4-mediated Gal-1 also regulated migration and invasion in SKOV3 cells. Interestingly, pharmacologic inhibition of PI3K signaling blocked Gal-1 secretion in LPS stimulated SKOV3 cells. Based on these data, the investigators speculated that TLR/PI3K induced Gal-1 promoted the migratory and invasive capacity of ovarian cancer cells and potentially important to mediating ovarian cancer metastasis [76].

### 2.4. Clinical Implication of Gal-1 as a Biomarker

Chen et al. [84] attempted to discern whether serum levels of Gal-1 could be used as a diagnostic for high-grade epithelial ovarian cancer. Differences in Gal-1 levels were detected via enzyme-linked immunosorbent assay (ELISA) in a pilot study assessing serum from healthy volunteers, patients with benign gynecologic tumors, patients diagnosed with epithelial ovarian cancer, or patients known to have another type of gynecologic cancer. Unfortunately, there was no difference in Gal-1 levels between healthy normal individuals, benign gynecologic tumor patients, and patients with high-grade epithelial cancer. Moreover, there was no difference in the levels of Gal-1 among the different histologies. There was, however, a difference among patients with non-metastatic epithelial cancer compared to metastatic disease [84]. Of interest, a subset of patients diagnosed with epithelial ovarian cancer had their blood drawn two days before and after debulking surgery. Nine of the 10 cases demonstrated a decrease in Gal-1 levels.

These same investigators compared Gal-1 and CA-125 levels. In their cohort, 98 of the 140 patients identified as positive by CA125 were also positive for using Gal-1. A subsequent co-immunoprecipitation study found that CA125 was associated with a labeled Gal-1, supporting a previous report that CA125 might serve as a receptor for Gal-1 in HeLa cells [85].

A more detailed analysis of the stroma associated with the ovarian carcinoma cells revealed that it was positive for Gal-1 in a majority of the samples scored. This is in contrast to the low to no positive Gal-1 staining in stroma found in normal ovarian tissues. It was further determined that the cancer-associated stromal staining was much higher in the invasive carcinoma when compared to non-invasive carcinoma. This was not the case when they assessed the Gal-1 in the cancer cells alone. In addition, the investigators revealed that the levels of Gal-1 in the cancer-associated stroma were positively correlated with stage. Furthermore, those patients with lymph node metastasis were found to have higher levels of Gal-1 stromal staining. If the cohorts were divided into weak and strong stromal staining, there was a correlation with the strong staining stroma and a recurrence rate in three years.

Although overall survival (OS) did not show a correlation with the levels of Gal-1 in the nucleus of ovarian cancer cells, it was reported that the levels of Gal-1 in the cytoplasm were closely related [86]. In addition, they showed, via a multivariate analysis, that interstitial Gal-1 was an independent prognostic factor in ovarian cancer patients. Together, these results suggest Gal-1 has the potential to be prognostic or a marker of disease progression in epithelial ovarian cancer.

The expression, localization, cellular distribution, proposed function and biomarker relevance for Gal-1 and other galectins in ovarian cancer discussed herein are summarized in Table 1.

## 3. Galectin 3-LGALS3

### 3.1. General Function of Gal-3

The *LGALS3* protein is one of the more well studied of the galectin family members. It is the only chimera galectin found in vertebrates [2,95,96,97,98,99]. Similar to other galectins, its expression is found among several types of cells. It is involved in a broad range of physiological and pathological processes, including, but not limited to, cell adhesion, invasion, proliferation, cell cycle, metastasis, and apoptosis [8,96,100,101,102,103].

**Figure 3:**
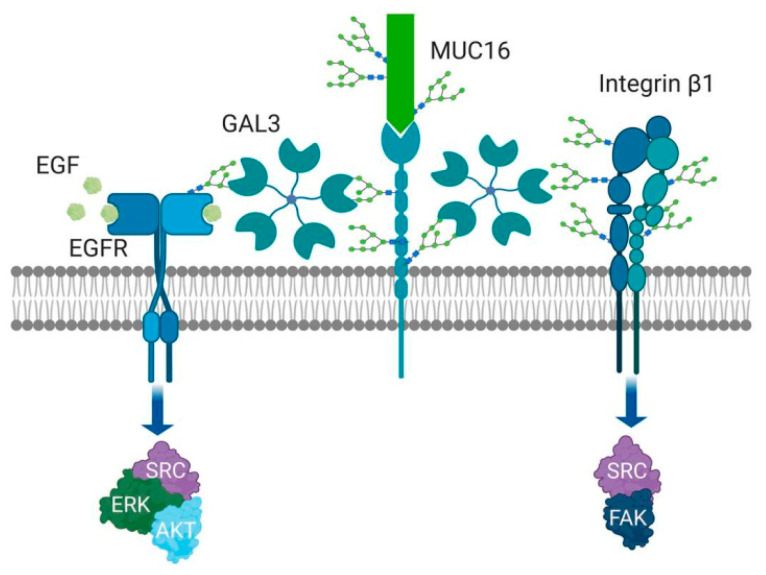
Gal-3 is involved in cell adhesion regulation, migration, invasion, angiogenesis, and metastasis. The specific extracellular Gal-3 function depends on the polymerization of Gal-3 into pentameric complexes. The action of Galectin 3 depends on glycan binding partners. These complexes link to glycans of high complexity (e.g., N-glycosylation lactosamine tetra-antennary forms and the Thomsen–Fredenreich antigen on O-glycans, especially in cancer. Through carbohydrate binding and polymerization to pentamers, Gal-3 forms a lattice and regulates the position of growth factor receptors, including EGFR, integrins and proteins like MUC16.

Galectin-3 has a unique complex structure that contributes to its versatile roles. In the absence of ligand, the N terminus and C terminus are loosely bound via low-affinity binding block the carbohydrate-binding domain. A high-affinity ligand (i.e., lactosamine) can outcompete the low-affinity binding of the N terminal domain and bind to the CBD. This enables the N terminal domain to become available for homo-pentamerization. These pentameric structures allow for the binding of other glycosylated proteins (see Figure 3 [104]). The ovarian cancer effects of Gal-3 are dependent on common glycovariants. The affinity of the CRD for Gal-3 favors binding to complex, tri and tetra-antennary forms found on complex N-glycans, dependent on MGAT-5 action [97]. Recent observations have identified high affinity binding between the common cancer antigen Gal(β1–3)GalNAc(α1-O-Ser/Thr (Thomsen–Friedenreich antigen,) and Galectin-3, which appears to enhance metastatic behaviors [105].

Intracellularly, Gal-3 is primarily found in the cytosol, but can also be found perinuclear, in the nucleus and near the mitochondria membranes [106,107]. Galectin-3 is also found in the extracellular space [96,108,109,110], whereby it can interact with a multitude of different binding partners. Its exit from the cell is believed to be due in part to exosomes and not via the more classic method of secretion from the endoplasmic reticulum or Golgi apparatus [111]. Once at the membrane surface, Gal-3 binds primarily polylactosamine-rich molecules located within the extracellular matrix or on the cell surface, where it can modulate functional properties (i.e., invasion, migration, and metastatic potential, etc.) that contribute to the tumor pathology/progression.

The forms Gal-3 can take are dependent on its location. Extracellularly, Gal-3 binds glycans of glycoproteins or glycolipids, via its carbohydrate recognition site at the C terminal domain, resulting in cross-linking, which is thought to be mediated by its N-terminal noncarbohydrate-binding domain [96]. This self-association can result in the establishment of pentameric structures [104]. Through the complex binding of the Gal-3 pentamers, lattices are formed that can regulate the special position and interactions of growth factor receptors, including epidermal growth factor receptor (EGFR), 1-integrin receptors, N-cadherin and cytotoxic T-lymphocyte associated factor-4 (CTLA-4), among others, in a variety of cell types [66,109,112,113,114]. Consequently, these complexes are postulated to regulate complex cell functions, including cell signaling, glycoprotein trafficking and cell–cell adhesion [97,98,115].

Apoptosis, one of many forms of programmed cell death, is mediated in large part by B cell lymphoma-2 (Bcl-2) protein family members [116,117,118]. The Bcl-2 protein family harbors Bcl-2 homology (BH) domains that mediate their interactions and execution of pro- or anti-apoptosis activity [116,117,118]. It is well understood that anti-apoptotic protein family members bind the BH3 domain of pro-apoptotic proteins like Bax and promote their oligomerization and subsequent pro-death activity at the mitochondrial level. In response to overwhelming cellular stress, whether it be endogenous or exogenous, the balance can shift in favor of the oligomerization of pro-apoptotic family members (i.e., Bax/Bax homodimers), leading to permeabilization of the outer mitochondria membrane, resulting in the release of cytochrome C (Cyt C). The release of Cyt C from the mitochondria triggers an enzymatic cascade orchestrated by members of the Caspase family, resulting in the ordered cleavage of DNA [116,117,118].

**Figure 4:**
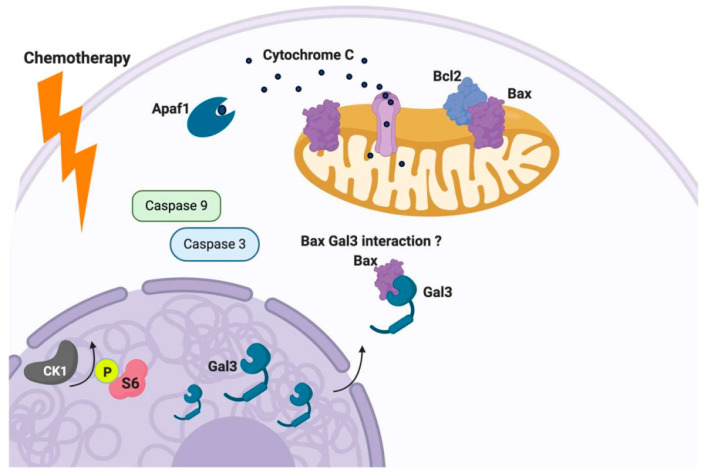
Galectin-3 as an inhibitor of the apoptotic response. Gal-3 can translocate from the cytosol and/or the nucleus to the mitochondria, inhibiting stressors of the mitochondrial membrane potential and subsequent release of Cyt C. In response to apoptotic stimuli, Gal-3 can translocate to the mitochondria and interact with Bax and prevent its function, as well as other pro-apoptotic Bcl-2 family members, subsequently preventing the formation of pro-death promoting homodimers.

Galectin-3 has been proposed as an inhibitor of the apoptotic response. Galectin-3 can translocate from the cytosol and/or the nucleus to the mitochondria, inhibiting the stressor induced disruption of the mitochondrial membrane potential and subsequent release of Cyt C [107,119]. Galectin-3 contains the anti-death aspartate-tryptophan-glycine-arginine (NWGR) motif, which is conserved in the BH1 domain of the Bcl-2 protein family. The NWGR motif within Gal-3 is located within the carbohydrate recognition domain (CRD). This motif is conserved in Bcl-2 protein family members, promoting balance by fostering the heterodimerization of pro and anti-apoptotic players (i.e., Bcl-2/Bax). In response to apoptotic stimuli, it has been shown that nuclear or cytoplasmic Gal3 can translocate to the mitochondria and interact with Bax in human thyroid carcinoma cells [102] (see Figure 4). This interaction of Bax and Gal-3 was confirmed by immunoprecipitation experiments and was only evident in response to doxorubicin [102]. The Gal-3 Bax interaction could be disrupted by GCS 100 (modified citrus pectin, a Gal-3 antagonist. These findings provide additional support for the concept that mitochondrial translocation of Gal-3 serves to tie up Bax and possibly other pro-apoptotic Bcl-2 family members, preventing them from forming pro-death promoting homodimers that initiate the cell death cascade.

### 3.2. Galectin-3 Expression, Localization and Relevance as a Biomarker of Ovarian Cancer 

Using immunofluorescence and a TMA, Labrie et al. [87] showed that Galectin-3 was present in both the epithelial and stromal cells of the normal ovary. Again, using another TMA, they showed that Gal-3 was commonly found in all the histological subtypes assessed, including serous, endometrioid, mucinous, and clear cell. When focusing on the serous subtype alone, there was Gal-3 positive staining present in epithelial cells (~60%) and stromal cells around the tumor (~40%) in the samples assessed. They found no association between epithelial or stromal Gal-3 with stage, recurrence, or death.

Wang et al. [120] conducted a meta-analysis of 36 eligible studies, looking at the prognostic role of Gal-3 expression in patients with solid tumors. The result of their meta-analysis was that there was a significant association of Gal-3 expression with OS in ovarian cancer.

### 3.3. Function of Gal-3 in Ovarian Cancer

There is sufficient data to support the role of ovarian cancer stem cells in the repopulation of the recurrent tumor, contributing to the heterogeneity of the disease, and chemoresistance [121,122]. More recently, there have been reports implicating that Gal-3 supports the stemness phenotype in different malignancies [103,123]. This is not surprising, given that overexpression of Gal-3 can promote properties associated with stemness, including chemoresistance [31,89,90,123], enhanced sphere or colony-forming capacity [88], enhanced DNA damage response [124], increased tumorigenesis [125], etc. Consequently, Kang and colleagues [88] assessed multiple ovarian cancer cell lines for their capacity to form spheres. Of the cell lines tested, A2780, OVCAR3, OVCAR429, SNU-251, and SKOV3 were proficient in forming tumorspheres. The SKOV3 and OVCAR429 lines had high levels of Gal-3, and the A2780 and OVCAR3 were considered to have low levels of Gal-3. Using a shRNA strategy, they depleted Gal-3 in the high Gal-3 cells SKOV3 and OVCAR429. This resulted in a reduced number of spheres, and those spheres that developed were smaller in size when compared to the control shRNA treated cells. Moreover, they reported that the total number of cells that could form spheres was reduced as well. 

To assess the impact of increased Gal-3 levels, they transformed A2780 and OVCAR3 (low Gal-3 level lines) with Gal-3 containing plasmids. This resulted in an increase in the number of spheres, as well as the number of cells within a sphere. Likewise, the rise in Gal-3 levels corresponded with an increase in the number of cells that could form spheres. Of interest, the levels of CD133, which is a cell surface stem cell marker in ovarian cancer, increased in Gal-3 overexpressing A2780 cells. In addition, the levels of CD133 and Gal-3 were increased after OVCAR3 cells were cultured in sphere-forming conditions. Based on these data, the investigators surmised that Gal-3 contributed to the stem-like properties of ovarian cancer cells [88].

The shRNA induced reduction of Gal-3 corresponded with a decrease in cell viability, and the increase level of Gal-3 was associated with an increase in cell viability. The A2780 cell line with elevated levels of Gal-3 was more resistant to cisplatin than their controls. The effect of increased Gal-3 levels on the OVCAR3 cells was not as pronounced, but there remained some level of resistance to cisplatin or paclitaxel, albeit at specific concentrations. This finding was similar to previous studies which demonstrated that treatment resistance was concurrent with an increase in Gal-3 levels in ovarian cancer cells [31,123].

The shift in Gal-3 levels also corresponded with the cells’ invasive and migratory properties. The elevated Gal-3 levels equated to increased invasion and migration relative to their respective control cells, as determined by transwell and migration assays. The inverse was seen with the cells that underwent shRNA reduction in Gal-3 levels. To extend their in vitro findings, utilizing a xenograft nude mouse model of ovarian cancer, Kang et al. [88] demonstrated that the A2780 cells overexpressing Gal-3 had a larger tumor volume than their control counterparts, further supporting the argument that Gal-3 contributes to the overall pathology of ovarian cancer [88].

It has been reported that Gal-3 exerts its pro-tumor promoting properties through the Thomsen–Friedenreich (TF) antigen [126,127], which occurs in 90% of all human cancer cells [128]. The TF antigen is clearly expressed in most ovarian carcinomas, with minimal expression in benign and normal ovarian tissues [129]. In ovarian cancer, mucins MUC1, MUC5AC, MUC6 and MUC16 are carriers of TF antigen [130,131,132]; among them, MUC1 is a natural ligand for Gal-3. The binding of Gal-3 to MUC1 can trigger a cascade of transmembrane signaling events. For example, in SKOV3 cells, Gal-3 binding activates the MAPK and PI3K/Akt signaling pathways, leading to the enhancement of cell proliferation and motility [133]. In non-ovarian tumor cell types, the binding of Gal-3 to cancer associated TF/MUC1 induces redistribution of MUC1 on the cell surface, increasing cancer cell homotypic aggregation and the formation of tumor micro-emboli [134]. It also promotes metastasis by enhancing cancer cell endothelial adhesion [135,136]. These latter properties have yet to be shown in ovarian cancer.

## 4. Galectin 7 (LGALS7)

### 4.1. General Function of Gal-7

Galectin-7 is a prototype member of the galectin family that is encoded by the gene *LGALS7* on chromosome 19q13.2 [137]. Galectin-7, acting intracellularly or extracellularly, is involved in a variety of processes, including epithelial maintenance, cell adhesion, cell migration, and apoptosis [137,138,139].

### 4.2. Galectin-7 Expression, Localization and Relevance as a Biomarker of Ovarian Cancer

Kim et al. [91], evaluated the levels of Gal-7 and compared those levels with clinicopathological variables and survival outcomes, in a small cohort of paraffin-embedded samples derived from patients that were diagnosed with ovarian cancer. There were 63 samples of ovarian cancer with mixed histologies and five normal ovarian samples obtained from patients diagnosed with benign gynecologic disease. The authors reported week positive cytoplasmic and nuclear staining evident in the epithelial cells of the normal ovary. In contrast, much higher levels of Gal-7 were observed in the malignant samples. The cohort of malignant samples was divided into two groups (high vs. low), based on their immunohistochemical score. There was no difference with respect to stage, grade, histological type, and sensitivity to treatment among the two groups. However, patients with higher levels of Gal-7 had a more inferior OS; 72 months for patients with low levels of Gal-7 vs. 56 months for those with high levels of Gal-7. Using a multivariate analysis, they determined that advanced stage, platinum resistance, and high Gal-7 expression was consistently an independent prognostic factor for poor OS in their cancer patients. Labrie et al. [92] also evaluated Gal-7 in a TMA, which contained a total of 112 patient samples, including normal, benign serous and mucinous, borderline serous, and mucinous, serous, endometrioid, transitional cell, clear cell, and granular tumors. They found no detectable levels of Gal-7 in the normal ovarian tissue. However, they did find Gal-7 positive staining in the epithelial cells of all the ovarian cancer subtypes. Within this TMA, Gal-7 was found mostly in the cytoplasm of the tumor cells. They found no correlation with levels of Gal-7 and the age of the patients of stage of disease. They did find that Gal-7 was present more frequently in metastatic samples compared to non-metastatic samples.

Moreover, Gal-7 levels were elevated in high-grade, borderline, and metastatic samples relative to benign tumors. The low-grade tumors also had lower levels than samples representing metastatic tumors. These same investigators also analyzed the public RNAseq datasets obtained from the cBio Cancer Genomics Portal (http://cbioportal.org), which revealed a correlation between *LGALS-7* mRNA and a lower OS with ovarian serous cystadenocarcinoma.

In the subsequent report by Schulz et al. [86], Gal-7 was found to be primarily present in the cytoplasm of the tumor cells, with just a few cases showing nuclear positivity. The level of expression varied among the 129 cases showing positivity, out of a total of 149 specimens. There were 15 samples considered to have high Gal-7 levels, 114 considered to have low levels of Gal-7, and 20 that were negative. Within the different subtype, the serous ovarian histological type had the highest levels of Gal-7, whereas the endometrioid had the weakest staining. There was a reduced OS for cases with high positivity for Gal-7 and a better OS rate for Gal-7 negative cases, when compared to cases with low expression of Gal-7. Their multivariate analysis supported the concept that higher Gal-7 levels could be an independent prognostic factor for OS in ovarian cancer. In general, these studies collectively imply that increased levels of Gal-7, as determined by immunohistochemistry, imply a poor outcome.

Regarding the localization of Gal-7, there are reports that it is detected mainly in the nucleus of tumor cells [91], reports that it is localized in the cytoplasm and extracellular compartment [92], and reports that it is present only in the cytoplasm in a cohort of primary ovarian cancer samples [86], as evidenced by immunohistochemistry.

### 4.3. Gal-7 Function in Ovarian Cancer

Kim et al. [70] evaluated Gal-7 levels in Hey8A, Hey8A-MDR, SKOV3ip1, SKOVTR, A2780-PAR, and A2780-CP20 cell lines by Western blot analysis. Although not quantified, qualitatively, the Hey8A, A2780-PAR, and SKOV3ip1 demonstrated higher levels when compared to the SKOVTR, Hey8A-MDR, and A2780-CP20 cell lines. Examining the differential proliferation rates between the A2780-PAR line and the A2780-CP20 line, the A2780-PAR line, with higher levels of Gal-7, proliferated at a much higher rate than the A2780-CP line, which displayed lower levels of Gal-7. The reduction of *LGALS-7* by siRNA strategy resulted in a decrease in mRNA and protein, which was concurrent with a decreased cell proliferation.

Labrie et al. [92] highlighted the contrasting roles of p53 mediated Gal-7 effects in different subtypes. Given these deferential effects, they assessed Gal-7 levels in cell lines with differential p53 status. They showed that OVCAR-3 cells, which harbor a mutated p53, express Gal-7. However, they did not detect Gal-7 in A2780 and COV434, which have wild type p53, or in SKOV3 cells with a p53 null genotype. To test whether forced expression of a mutant p53 could alter LGLAS-7 mRNA and protein levels, they transfected SKOV3 and OVCAR3 cells and found that there is a concurrent increase in LGLAS-7 mRNA and protein levels, with the increase in p53 mRNA and protein levels. Likewise, the suppression of an endogenous p53 mutant by a siRNA diminished *LGALS7* levels, as determined by RT-PCR.

While Labrie and colleagues [92] found most of the immunohistochemistry based Gal-7 primarily in the cytoplasm of the tumor cells, they also found Gal-7 in the supernatants derived from both OVCAR-3 and in SKOV3 cells transfected with human Gal-7 expression vector, suggesting that it is released from the cell, either actively or passively. The levels of Gal-7 in the supernatant were determined by both Western blots and ELISA. Moreover, they used confocal microscopy to show that Gal-7 was present and that Gal-7 was bound to the cell surface and in the cytoplasm in OVCAR3 and SKOV3 cells. The investigators added -lactose to the culture media, which resulted in the decreased binding of Gal-7 to SKOV-3 cells, suggesting that the binding might be CRD-dependent. They further assessed Gal-7 binding activity in the A2780 and SKOV3 cells using a FITC-labeled recombinant Gal-7 and flow cytometry. Again, the addition of -lactose decreased the binding of Gal-7. Collectively, they surmised that Gal-7 was present intra- and extracellularly.

Based on the finding that Gal-7 was found extracellularly, they went on to demonstrate that Gal-7 was released from culture ovarian cancer cells. For this, they again used the FITC- labeled recombinant Gal-7 and found it bound to the surface of Jurkat T cells in a dose-dependent manner. The binding was inhibited by the addition of -lactose, as well as an unlabeled Gal-7. The increase in binding was found to be associated with an increase in apoptosis of the T cells. They confirmed this effect in human PBMC, as evidenced by an increase in apoptosis in CD-14-positive monocytes and CD4- or CD8-positive T cells. Together, these findings support the idea that Gal-7 may have immunosuppressive effects.

## 5. Galectin-8 (LGALS8)

### 5.1. General Function of Gal-8

Galectin-8 is one of the tandem repeat galectins that contain two CRDs connected by a linker chain [140,141]. Galectin-8 is expressed in several tissues, including the lung, liver, kidney, brain, and myocardium [142]. It has been suggested that it mediates angiogenesis-related disorders of myocardial infarction, diabetic retinopathy, and various cancers, by promoting endothelial cell migration and tubular formation and regulating angiogenesis [143]. It has also been shown to induce cell arrest and apoptosis in Jurkat T cells [144].

### 5.2. Gal-8 Expression and Localization in Ovarian Cancer

Given that Gal-8 has received relatively little attention as a prognostic marker in ovarian cancer, Schulz and their team [93] used the human protein atlas (www.proteinatlas.org) to conduct an in silico analysis of Gal-8 expression in normal and malignant ovarian tissue. Galectin-8 was not detected via antibody staining in ovarian stromal cells. However, 8 of 12 ovarian cancer tissues had what was deemed medium Gal-8 expression. Based on these preliminary findings, Schulz and colleagues [93] chose to evaluate 156 ovarian cancer samples using immunohistochemistry and compared the Gal-8 levels and its localization with clinical and pathological outcomes. Of the 156 ovarian cancer samples, they were able to evaluate 143 samples for Gal-8. These samples were of mixed histologies, with serous making up the bulk of the population (n=102). The remainder were of clear cell, endometrioid and mucinous subtypes. Galectin-8 positive staining was predominantly in the cytoplasm and nuclei of ovarian tumor cells. Galectin-8 positivity was not evident in the peritumoral stroma. High Gal-8 staining in the cytoplasm was evident in 67% of the samples, while 32% of the samples had low levels. They found that low Gal-8 expression in the cytoplasm correlated with lymph node metastasis, as well as higher stage. Half of the samples had Gal-8 positive nuclei. It was determined that positive nuclear Gal-8 staining was seen more often in stage I and II disease. 

Based on Kaplan–Meier analysis, those patients with high Gal-8 levels had a better DFS and overall survival. There was no difference in DFS and overall survival when comparing nuclear Gal-8 levels. Finally, a multivariate analysis revealed that Gal-8 positive staining served as a prognostic factor, independent of clinical and pathological variables.

Labrie et al. also utilized a TMA for analysis of galectin expression in normal and diseased tissue [87]. Using immunofluorescence in a TMA constructed from normal ovarian and fallopian tubes tissue samples, they found that Gal-8 was present in both the epithelial and stroma of normal ovarian and fallopian tube tissue samples. The level of Gal-8 positive staining was much more evident in the tube than in the stroma. For the most part, the Gal-8 positive staining was present in the cytoplasm.

In a second TMA described above, they evaluated the samples from 63 patients, representing serous, endometrioid, mucinous, and clear cell histology, and Gal-8 was commonly found in all histological subtypes. In yet another TMA representative of 209 specimens of ovarian high-grade serous cancer, approximately 55% of the samples stained positive for Gal-8 in the epithelial portion of the tumor, and roughly 30% stained positive for Gal-8 in the stromal area immediately adjacent to the tumor. Of interest, a univariate Cox analysis of the Gal-8 positive staining revealed that epithelial Gal-8 was correlated with chemoresistance [87], suggesting that the presence of Gal-8 may have some prognostic value.

The differing results observed in the studies describing the presence and locality of Gal-8 could be attributed, in part, to the technique or the affinity of the different antibodies used. Moreover, there are reportedly seven different isoforms of Gal-8 encoded by the *LGALS8* gene as a result of alternative splicing [145]. There are no reports of specific antibodies targeting any particular isoform of Gal-8. Therefore, only the total expression of the reactive Gal-8 isoforms is observed by immunohistochemistry. It is also not clear whether different anti-Gal-8 antibodies have a high affinity for a particular Gal-8 isoform [93]. Future functional studies may discern whether the isoforms display different actions.

### 5.3. Relevance of Gal-8 as a Biomarker for Ovarian Cancer

Labrie et al. [87] assessed the plasma levels of Gal-8 in 160 samples from healthy controls and 145 from patients diagnosed with ovarian cancer. They observed that Gal-8 plasma levels were significantly higher in patients with high-grade serous cancer (HGSC), as compared to the plasma from healthy controls. High Gal-8 plasma levels were associated with a lower 5-year disease-free interval and OS. High plasma Gal-8 levels were predictive for 5-year DFS and 5-year OS in patients with low cancer antigen 125 (CA125). The authors concluded that high Gal-8 might be a powerful molecular marker as a prognostic predictor of HGSC.

## 6. Galectin-9 (LGALS9)

### 6.1. General Function of Gal-9

Galectin-9 protein is transcribed from the *LGALS9* gene, which is encoded on the short arm of chromosome 17 [146]. Gal-9 has been reported to have a number of biological functions, including contributing to innate and adaptive immunity [147]. Moreover, it has been shown to contribute to tumorigenesis and tumor pathology, by promoting cell transformation, cell cycle regulation, cell adhesion and angiogenesis [21,148,149]. Galectin-9 has been evaluated histologically and as a prognostic marker for several cancer types, including gastric cancer, non-small cell lung cancer, hepatocellular carcinoma, melanoma, and breast cancer [29,149,150,151,152].

### 6.2. Gal-9 Expression and Localization in Ovarian Cancer

Similar to Gal-8, Gal-9 was found to be present in a TMA containing normal ovary and fallopian tube samples [87]. Compared to Gal-8 positive staining, the cytosolic staining for Gal-9 was weaker. Again, using immunofluorescence and another TMA representing serous, endometrioid, clear cell, and mucinous subtypes, it was observed that unlike Gal-8, which was found in most ovarian cancer subtypes, Gal-9 was not as readily prevalent in all subtypes. Interestingly, Gal-9 was found to be present in more than 80% of all clear cell and mucinous samples; whereas, Gal-9 was evident in less than 40% of the HGSC samples in that TMA. Using a larger TMA, which had 209 samples of HGSC, they showed that roughly 65% of the tumors had Gal-9 positive staining in the stromal compartment of the tumor. In contrast, only about 45% of the tumor cells stained positive for Gal-9. The staining was reportedly strong in the cytosol. More interesting, however, was that the tumor cells that stained positive for cytosolic Gal-9 often showed evidence of positive staining in aggregates in the perinuclear region. Based on other immunohistochemical stains, they determined that these aggregates were not autophagosomes of mitochondria. Galectin-9 was commonly found in the stroma immediately adjacent to tumor cells.

With regards to clinical correlates, a univariate Cox analysis showed that epithelial Gal-9 punctum staining correlated with a lower 5-year survival rate. The Cox proportional hazards model showed that epithelial Gal-9 in the punctum was independently linked to a poor 5-year OS. There was no association when assessing stromal Gal-9. These same investigators [87] also showed that epithelial Gal-9 had a significant predictive value of 5-year OS in patients with low plasma levels of CA125, but not with high plasma levels of CA125.

In contrast to Labrie and colleagues, Schulz et al. [93] reported that Gal-9 positive staining was primarily evident in the cytoplasm of ovarian cancer cells, but not in the nucleus or peritumoral stroma. In their cohort of 147 ovarian cancer samples, the high level of Gal-9 was related to a lower grade of the tumor, lower stage, and patients of a younger age. Furthermore, the majority of Gal-9 negative cases showed high-grade, advanced stage, and older age. In addition, cases with moderate Gal-9 expression showed a reduced PFS and decreased OS compared to Gal-9 negative cases.

The differences observed in the studies described, with respect to levels and expression of Gal-9 and their clinical correlates, do not provide a great deal of confidence in their overall value. The functional data is likely more reliable as a predictor of outcomes.

### 6.3. Gal-9 Function in Ovarian Cancer

Functionally, Galectin-9 (Gal-9) has been implicated in regulating apoptosis in various types of cancer cells [153,154,155]. *LALS9* was first described as being present in OVCAR3 cells by RT-PCR, albeit inconsistently [156]. Jafari et al. [94] went on to assess the anti-tumor effect of Gal-9 in the OVCAR3 cell line. They demonstrated that Gal-9 inhibited cell proliferation in a dose-dependent manner, as evidenced by an MTT assay. There was also a Gal-9 dose-dependent increase in intracellular levels of reactive oxygen species, which was concurrent with an increase in Caspase 3 and Caspase 6 activity. The addition of Gal-9 also resulted in a reduction in mitochondrial membrane potential (ΔΨm) in the OVCAR-3 cells. Moreover, there was a decrease in Bcl-2, which was inversely associated with a reduction of Bax. Collectively, these findings support their idea that an increase in Gal-9 would push OVCAR3 cells towards cell death via apoptosis. Furthermore, this effect is mediated by mitochondria. Overall, the functional data would suggest Gal-9 would likely serve to oppose Gal-3’s anti-apoptotic.

### 6.4. Relevance of Gal-9 as a Biomarker of Ovarian Cancer

Labrie et al. initially assessed the plasma levels of Gal-9 in a small cohort of ovarian cancer patients (*n* = 35) by ELISA. Gal-9 plasma levels were elevated in blood from malignant cases compared to the healthy controls. Therefore, as described above for Gal-8, they went on to measure Gal-9 levels in a larger cohort by ELISA. Their first finding was that plasma levels did not necessarily correlate with tissue levels in primary tumors. However, high plasma levels of Gal-9 were associated with a lower 5-year DFS and 5-year OS, and in the case of low plasma levels of CA125, Gal-9 was only associated with 5-year DFS [87]. A multivariate analysis showed that plasma levels of Gal-9 was an independent predictor of 5-year DFS and 5-year OS. The authors suggested that galectin levels in plasma, as well as the expression in tumor and peritumoral stromal cells, could potentially be used to predict 5-year DFS, chemotherapy response, and 5-year OS in HGSC patients and to potentiate the predictive value of CA125 [87].

Collectively, these studies share disparate findings with respect to levels, distribution, and outcomes; whether or not this is attributed to the different several splice variants that were previously reported [26,157] or differences in technique, antibodies or types of analyses remains to be determined.

## 7. Therapeutic Targeting of Galectins

### 7.1. General Strategies for Disrupting Galectin Mediated Effects

As galectins play a pivotal role in the development of a variety of human diseases, blocking the effects of tumor promoting galectins has become a rational target for therapeutic applications, particularly Gal-1 and Gal-3. Novel inhibitors of both Gal-1 and Gal-3 have been developed, utilizing a variety of strategies, including blocking the CRD using oligosaccharides, small molecules, short hairpin RNA (shRNA), or monoclonal antibodies, with a varying degree of clinical success. The various sites of galectin action make the strategies for targeting potentially very different. The inhibition of protein glycosylation by inhibition of the glycosylation enzymes (such as inhibition of the key N-glycosylation enzyme MGAT V) can be highly effective, involving the reduction of both intracellular (apoptosis, nuclear effects, etc.) and extracellular functions (binding to N-glycosylation sites on key proteins) [158,159]. Such an intervention would necessarily affect all galectin functions and might lack specificity. In contrast, long-acting protein inhibition by truncated binding domains or monoclonal antibodies would be restricted to extracellular functions and might miss key functions. Small molecule inhibitors may have variable cellular uptake and could also penetrate the central nervous system, with potential effects that would be unlikely in antibody therapeutics [160].

### 7.2. Specific Targeting of Galectins in Cancer

Exploiting the CRD of galectins and their preference for binding to galactose residue, the oligosaccharides GM-CT-01, GR-MD-02 (both are galactomannan polysaccharide that present N-terminal galactose residue), GCS-100 (a modified citrus pectic carbohydrate), and TD139 (bis-3-deoxy-3-[4-(3-fluorophenyl)-1H-1,2,3-triazol-1-yl]-b-d-galactopyranosyl[1]sulfane) have shown pre-clinical efficacy in diseases that are galectin mediated, including liver fibrosis, melanoma, myeloma, and lung fibrosis [161,162,163,164]. While GM-CT-01 has shown promise in early phase clinical trials in colon cancer [165], GR-MD-02 failed to improve fibrosis or liver-related outcomes in patients with nonalcoholic steatohepatitis [166]. TD139 has also shown promise in the treatment of idiopathic pulmonary fibrosis through its suppression of Gal-3 levels [167]. While these early results are promising, oligosaccharide derivates are broad-spectrum, often targeting more than one galectin, and can potentially result in undesirable side effects. 

Small molecule galectin inhibitors have been employed as treatment strategies against galectin-mediated tumorigenesis. OTX008 is a phenyl-based molecule that binds the CRD of Gal-1 and has been shown to inhibit tumor cell survival and angiogenesis in ovarian cancer cell lines and synergizes with chemo- and immunotherapies in vitro [168,169]. Other galectin inhibiting small molecules include 6DBF7, a dibenzofuran (DBF)-based peptidomimetic of the Gal-1, which has been shown to block tumor angiogenesis and tumor growth in melanoma, lung, and ovarian cancer mouse models [170]. GB1107 (3,4-dichlorophenyl 3-deoxy3-[4(3,4,5-trifluorophenyl)-1H-1,2,3-triazol-1-yl]-1-thio-a-Dgalactopyranoside) is a small molecule Gal-3 inhibitor that has been shown to reduce human and mouse lung adenocarcinoma cell growth [171]. Clinical data for either 6DBF7 or GB1101 have not been reported to date.

Other galectin inhibiting strategies employed include shRNA and anti-galectin monoclonal antibodies. Li et al. achieved Gal-3 knockdown in vivo using a Gal-3 short hairpin RNA expressed in the pLKO.1 lentiviral vector [172]. The survival of animals was significantly improved with the Gal-3 inhibition treatment. To date, there are no reports of utilizing this strategy in large mammals. Monoclonal anti-galectin-1 antibody (F8.G7) was shown to decrease tumor angiogenesis and promote tumor regression in a mouse model of Kaposi’s sarcoma [173].

Galectins have been recognized to play a vital role in an array of human diseases, and as such, have become the subject of interest as therapeutic targets in cancer therapies, as well as lung and heart diseases. A multitude of pharmacologic strategies have been undertaken to block the interactions between galectins and their saccharide partners. Some have shown promise in early clinical trials. Further research will help elucidate which galectin inhibition strategy works best in a clinical setting. Of importance herein there is sufficient evidence to suggest that disruption of pro tumor galectins may well be a potential strategy to explore for the treatment of highly aggressive ovarian cancer.

## 8. Conclusions

Our objective herein was to review what is known about distinct galectin family members in the context of ovarian adenocarcinoma, with emphasis on their levels, extra and intracellular localization, association with clinical and pathological features, implied functions, diagnostic or prognostic potential and strategies being developed to disrupt their negative actions. Of all the galectin family members, Gal 1, 3, 7, and 9 appear to be the most relevant to the invasive, metastatic, chemoresistance and immunosuppressive properties of ovarian tumors. While the functional significance in the different studies, for the most part, aligned with each other, their prognostic or diagnostic potential remains uncertain. It is likely that as more specific antibodies covering the various isoforms are developed, some of the discrepancies will be resolved. Nevertheless, the evidence provided thus far by the shRNA, siRNA and pharmacologic inhibitors suggests that targeting galectin mediated effects could serve to augment standard of care approaches.

## Figures and Tables

**Table 1:** Expression, localization, cellular distribution, proposed function, and biomarker relevance for galectins in ovarian cancer.

Galectin	Histological Subtypes	Distribution	Cellular Localization	Function in Ovarian Cancer	Prognosis
Gal-1	Serous Endometrioid Mucinous Clear cell [70,84,86]	Tumor cell Tumor stroma [69] Stroma [70]	Nucleus, Cytoplasm [70]	Mediate EMT, cell proliferation, migration, invasion, and cell signaling [71,76]	Higher levels of Gal-1 in the peritumoral stroma associated with poor PFS [70] Cytoplasmic levels of Gal-1 were closely related with OS [86] Interstitial Gal-1 was an independent prognostic factor in ovarian cancer patients [86]
Gal-3	Serous Endometrioid Mucinous Clear cell [87]	Cancer cell Stroma [86,87]	Cytoplasm [31,86,87] Nucleus [86]	Contributing to stem-like properties [88] Promoting chemoresistance [31,88,89,90] Promoting cell invasion and migration [88]	High Gal-3 cytoplasmic expression correlated with poor PFS [31] Low Gal-3 expression in nuclei associated with reduced OS [86] High Gal-3 expression correlated with reduced OS [90]
Gal-7	Serous Endometrioid Mucinous [91] Serous Endometrioid Mucinous Clear cell [86]	Epithelial cell [92]	Nucleus, Cytoplasm [91] Cytoplasm [86]	Down-regulation of Gal-7 expression inhibited tumor cell proliferation [91] Expression increased the invasive properties of ovarian cancer cells and by killing immune cells [92]	Higher levels had a more inferior OS [91] High expression reduced OS and higher expression is an independent prognostic factor for OS [86]
Gal-8	Serous Endometrioid Mucinous Clear cell [87,93]	Tumor cell [87,93] Stroma [87]	Nucleus [93] Cytoplasm [87,93]	None found	High expression in epithelial component correlated with chemoresistance [87] Positive nuclear staining correlated with lower stage [93] Low expression in cytoplasm correlated with lymph node metastasis and higher stage [93] Higher plasma levels correlated with lower 5-year DFS and 5-year OS [87] High expression in the cytoplasm correlated with better DFS and OS [93]
Gal-9	Serous Endometrioid Mucinous clear cell [87,93]	Cancer cell [87,93] Stroma [87]	Cytoplasm [87,93]	Inhibiting cell proliferation and pushing cells towards apoptosis [94]	High expression presented more often with low tumor stage, lower grading, and younger age [93] Epithelial expression correlated with a lower 5-year OS [87] High plasma levels were associated with a lower 5-year DFS and 5-year OS [87] High expression showed the more favorable PFS and OS [93]

## Abbreviations

AKT	Protein kinase-B
Bcl-2	B cell lymphoma-2
BH3	Bcl-2 homology 3
BrdU	Bromodeoxyuridine
CA125	Cancer antigen 125
CBD	Collagen-binding domains
CCK-8	Cell counting kit 8
CRD	Carbohydrate recognition domain
CTLA-4	Cytotoxic T-lymphocyte associated factor-4
Cyt C	Cytochrome C
DBF	Dibenzofuran
DDR	DNA damage response
DFS	Disease free survival
EGFR	Epidermal growth factor receptor
EMT	Epithelial mesenchymal transition
ELISA	Enzyme-linked immunosorbent assay
FITC	Fluorescein isothiocyanate
Gal-1	Galectin-1
Gal-11	Galectin-11
Gal-13	Galectin-13
Gal-14	Galectin-14
Gal-15	Galectin-15
Gal-2	Galectin-2
Gal-3	Galectin-3
Gal-5	Galectin-5
Gal-7	Galectin-7
Gal-8	Galectin-8
Gal-9	Galectin-9
HIV	Human immunodeficiency virus
HGSC	High grade serous cancer
IL-17	Interleukin-17
Il-2	Interleukin-2
JNK1	C-jun-NH2-terminal kinase 1
LPS	Lipopolysaccharide
MALP2	Macrophage-activating lipopetide-2
MET	Mesenchymal epithelial transition
MTT	3-(4,5-Dimethylthiazol-2-yl)-2,5-diphenyltetrazolium bromide
MUC1	Mucin 1
MUC5AC	Mucin 5AC
MUC6	Mucin 6
MUC16	Mucin 16
NF-IL6	Nuclear factor for interleukin 6
NWGR	Aspartate-tryptophan-glycine-arginine
OS	Overall survival
PBMC	Peripheral blood mononuclear cell
PFS	Progression free survival
PI3K	Phosphoinositol 3 kinase
shRNA	Small hairpin RNA
siRNA	Small interfering RNA
TG	Thomsen–Friedenreich (TF)
TLR	Toll-like receptor
TLR-3	Toll-like receptor-3
TLR-4	Toll-like receptor-4
TMA	Tissue microarray
Treg	T regulatory
